# Documenting malaria case management coverage in Zambia: a systems effectiveness approach

**DOI:** 10.1186/1475-2875-12-371

**Published:** 2013-10-25

**Authors:** Megan Littrell, John M Miller, Micky Ndhlovu, Busiku Hamainza, Moonga Hawela, Mulakwa Kamuliwo, Davidson H Hamer, Richard W Steketee

**Affiliations:** 1 PATH Malaria Control and Evaluation Partnership (MACEPA), Seattle, WA, USA; 2 PATH Malaria Control and Evaluation Partnership (MACEPA), Lusaka, Zambia; 3 Chainama Hills College Hospital, Lusaka, Zambia; 4 National Malaria Control Centre, Ministry of Health, Lusaka, Zambia; 5 Zambia Center for Applied Health Research and Development, Lusaka, Zambia; 6 Center for Global Health and Development, Boston University, Boston, MA, USA

**Keywords:** Malaria, Case management, Coverage, Health facility survey, Systems effectiveness

## Abstract

**Background:**

National malaria control programmes and their partners must document progress associated with investments in malaria control. While documentation has been achieved through population-based surveys for most interventions, measuring changes in malaria case management has been challenging because the increasing use of diagnostic tests reduces the denominator of febrile children who should receive anti-malarial treatment. Thus the widely used indicator, “proportion of children under five with fever in the last two weeks who received anti-malarial treatment according to national policy within 24 hours from onset of fever” is no longer relevant.

**Methods:**

An alternative sequence of indicators using a systems effectiveness approach was examined using data from nationally representative surveys in Zambia: the 2012 population-based Malaria Indictor Survey (MIS) and the 2011 Health Facility Survey (HFS). The MIS measured fever treatment-seeking behaviour among 972 children under five years (CU5) and 1,848 people age five years and above. The HFS assessed management of 435 CU5 and 429 people age five and above with fever/history of fever seeking care at 149 health facilities. Consultation observation and exit interviews measured use of diagnostic tests, artemisinin combination therapy (ACT) prescription, and patient comprehension of prescribed regimens.

**Results:**

Systems effectiveness for malaria case management among CU5 was estimated as follows: [100% ACT efficacy] x [55% fever treatment-seeking from an appropriate provider (MIS)] x [71% malaria blood testing (HFS)] x [86% ACT prescription for positive cases (HFS)] x [73% patient comprehension of prescribed ACT drug regimens (HFS)] = 25%. Systems effectiveness for malaria case management among people age five and above was estimated at 15%.

**Conclusions:**

Tracking progress in malaria case management coverage can no longer rely solely on population-based surveys; the way forward likely entails household surveys to track trends in fever treatment-seeking behaviour, and facility/provider data to track appropriate management of febrile patients. Applying health facility and population-based data to the systems effectiveness framework provides a cogent and feasible approach to documenting malaria case management coverage and identifying gaps to direct program action. In Zambia, this approach identified treatment-seeking behaviour as the largest contributor to reduction in systems effectiveness for malaria case management.

## Background

Malaria has been a pervasive scourge across the centuries, particularly in Africa, however tremendous advances in malaria control have been achieved in recent years [1,2]. The improvements have been most notably documented in the coverage of malaria prevention interventions and have led to marked declines in malaria-related child mortality. Despite this progress, malaria remains a leading cause of death among children under five [3], and a leading contributor to the global burden of disease, particularly in sub-Saharan African [4]. The estimated number of malaria cases in Africa in 2010 was 174 million [1]. Families, communities and health systems face an ongoing burden of managing malaria infections. The recently introduced highly efficacious artemisinin combination therapy (ACT) is now the first-line treatment in most countries where *Plasmodium falciparum* is endemic [1]. More recently, quality controlled rapid diagnostic tests (RDTs) have proven valuable in confirming infections and directing appropriate malaria treatment such that, along with microscopy, diagnostic confirmation of malaria is recommended worldwide [5].

Improvements in malaria control have been documented using population-based surveys to chart changes in intervention coverage [2,6]. These include the Demographic and Health Survey (DHS), the Malaria Indicator Survey (MIS), and the UNICEF Multiple Indicator Cluster Survey (MICS). Tracking progress through these surveys has been well characterized for malaria prevention coverage where standard indicators have been tracked over time and provide a reliable picture of improvements [2]. However, for case management, the primary indicator “proportion of children under the age of five years with fever in the last two weeks who received anti-malarial treatment according to national policy within 24 hours from onset of fever” [6] has several limitations. These include use of fever as a proxy measure for malaria infection, and reliance on caregiver reports for information on blood testing and medicines used for treatment [6]. Further, in the face of recent policy changes recommending confirmatory blood testing prior to treatment, presumptive treatment of fever is no longer a valid indicator for appropriate case management [7]. Routine health management information systems (HMIS) data capturing malaria blood testing, test results and treatments administered are not subject to these limitations. However, facility data fail to capture coverage among the substantial proportion of the population where treatment services are not available or among those who do not seek care within the public health system. Additionally, in many malaria-endemic countries, HMIS are weak, data are incomplete, contain inaccuracies, and are unavailable in an actionable timeframe [8]. Finally, HMIS data do not capture characteristics of an individual patient’s interaction within the health system. A facility-based survey designed to assess the extent and quality of service provision represents an approach that can provide complete and unbiased data in an actionable timeframe. Linking facility survey data with population-based data could provide a more complete picture of malaria case management coverage [8,9].

Zambia has made numerous improvements in malaria case management in the last decade including adopting the ACT artemether-lumefantrine (AL) as the first-line treatment for malaria in 2002, and introducing RDTs in health facilities in 2006 [10,11]. National guidelines for diagnosis and treatment were modified in 2009–10 to require parasitological confirmation by RDT or microscopy prior to malaria treatment wherever capacity is available [12]. Scale-up of blood testing and AL treatment was facilitated by substantial donor support. Between 2003 and 2010, external partners committed nearly $200 million to malaria prevention and control in Zambia [10]. Currently, the basic health care package offered through the public health system in Zambia includes blood testing and AL treatment. These and other basic health services are provided free of charge in health facilities located in rural and poor districts. Zambians access care at three basic levels of public health care: rural health posts and community outreach; urban and rural health centres; and primary, secondary and tertiary hospitals. Nearly all urban households are located within 5 km of a health facility as compared with 50% of rural households. The relatively small private for-profit sector operates in urban areas [13] and treatment seeking in the private sector for suspected malaria is relatively uncommon [14].

The MIS has been the primary tool for tracking recent malaria control progress in Zambia; four rounds have been implemented in 2006, 2008, 2010, and 2012. In 2011, a nationally representative Health Facility Survey (HFS) was implemented. 2011 HFS and 2012 MIS data and a systems effectiveness approach were used to document malaria case management coverage in Zambia following recent improvements. A systems effectiveness framework evaluates coverage by examining the necessary sequential components for effective health service delivery [15]. This framework documents the extent to which highly efficacious disease control tools effectively reduce disease burden. Effectiveness is a function of whether a disease control tool reaches the target group; users and providers comply and perform correctly; and a high level of coverage is sustained [16]. Effectiveness of highly efficacious anti-malarial drug (i.e. ACT) is a function of: 1) treatment-seeking behaviour among febrile individuals – seeking treatment from an appropriate provider; 2) malaria blood testing provided to patients with fever to confirm malaria infection; 3) appropriate treatment with ACT based on blood test results (provider compliance); and 4) patient adherence to anti-malarial treatment [17-20]. Consequently, the information required to use this approach must come from multiple sources, both from population data (using representative household surveys) and facility data (using representative health facility surveys). Benchmarking systems effectiveness can guide Zambia stakeholders towards action to improve malaria case management.

## Methods

### 2012 Malaria Indicator Survey (MIS)

Details on the Zambia MIS methods are described elsewhere [21]. Briefly, the MIS is a national population-based household survey designed to measure key indicators for tracking malaria control progress. A random sample of households stratified by urban and rural residence was drawn using two-stage cluster sampling. Standard enumeration areas were selected with probability-proportional-to-size at the first stage and a random sample of 25 households per cluster was selected at the second stage.

Within each sampled household, a standard questionnaire administered using a personal digital assistant (PDA), collected basic information about the household and each household member. The MIS Household and Woman’s Questionnaires are modeled after the survey instruments developed by the MEASURE DHS + programme and adopted and recommended for use by the Roll Back Malaria Monitoring and Evaluation Reference Group Task Force on Household Surveys [22]. The standard household roster was expanded to include questions about recent febrile illness, fever treatment-seeking behaviour, and anti-malarial treatment among all household members. Each woman between the ages of 15 and 49 completed a standard woman’s questionnaire interview that included questions about each of her children under age five. These questions included information about febrile illness occurring in the two weeks preceding the survey, treatment-seeking behaviour, blood testing, and anti-malarial treatment received for the fever. Where parental consent was obtained, children were administered an RDT and specimens were collected on a blood slide (BS) for microscopy [22].

Fieldwork was completed between April and May, 2012; 3,800 households participated in the survey. Within these households, information was collected in the household questionnaire on 16,928 household members, including 2,820 household members with recent fever. Information about 2,620 children under five was collected in the woman’s questionnaire from 2,301 biological mothers age 15–49, including 742 children with recent fever.

All MIS and HFS research activities were reviewed by the Research Ethics Committee of the University of Zambia on behalf of the Ministry of Health, the PATH Research Ethics Committee, and the US Centers for Disease Control Institutional Review Board. Final authority to conduct these research activities was obtained from the Ministry of Health.

### 2011 Health Facility Survey (HFS)

Details on the Zambia HFS methods are described elsewhere [23]. Briefly, the HFS was designed to provide nationally representative information on facility-based malaria case management. The study was modeled after the World Health Organization Integrated Child Health Facility Assessment [24] and applications in similar contexts [25].

The sampling frame consisted of a list of all health facilities in Zambia compiled in 2009 by the Ministry of Health (N = 1,843). Most health facilities in Zambia are government-run (85%), although a few are run by private for-profit (9%) or mission organizations (6%) [13]. As such, the sample represents health facilities in Zambia, with chance of including private and mission facilities equivalent to their representation among all national health facilities. The sample was drawn with explicit stratification from lists of all facilities at each level of care: health post (N = 266), urban health centre (N = 428), rural health centre (N = 1,042), and hospital (N = 107). Within each group, geographic stratification was done by ordering facilities by location (province, district), and a random sample of health facilities was selected using systematic random sampling. Forty-two facilities were sampled from each stratum for a total of 168 facilities. The overall facility response rate was 89% (N = 149). The selected sample was largely government (75%) and non-profit/mission facilities (11%); 12% of the sampled facilities were private for-profit and 3% were facilities for company employees (i.e., commercial farm or mining companies).

Each facility was surveyed for one day during which a sample of patients was selected. Inclusion criteria for the study were as follows: patients visiting the outpatient clinic for consultation with a health worker and presenting at the health facility for the current illness for the first time. There were 1,464 patients identified as eligible for the study. A participation rate of 95% (N = 1,394) was achieved. Of these eligible patients, consultation observation and exit re-assessment were performed for 1,290 patients who were reportedly seeking care for the illness for the first time. Of these patients, 872 had suspected malaria (self-reported fever or a history of fever and/or body temperature greater than or equal to 37°C), including patients seeking care at hospitals (N = 213), urban health centres (N = 221), rural health centres (N = 251), and health posts (N = 187). One-half (51%) of the patients with suspected malaria were under age five years.

Two patient-focused survey instruments were used: 1) consultation observation form to capture case management practices; and, 2) patient exit interview and re-examination. The silent consultation observation was completed first; laboratory tests and results for the patient were recorded on this form. After the patient fully completed the consultation, the exit interview was conducted to assess patient comprehension of the diagnosis and prescribed drug regimens as well as patient recall and assessment of health worker performance during the consultation. Finally, a patient re-examination performed by a trained health professional provided a gold standard malaria diagnosis (clinical assessment confirmed by RDT).

### Measurement and analysis

HFS indicators were created among the subsample of patients with suspected malaria. Suspected malaria was determined at an exit re-assessment and was defined as presence of self-reported fever or a history of fever and/or body temperature greater than or equal to 37°C. A variable was created to indicate whether or not these patients received a blood test during consultation according to the consultation observation form (interviewer observation and/or information recorded from patient records). Treatment with the national first-line ACT (AL) was measured at exit interview by review of patient medicines and/or prescriptions obtained during the facility visit. Variables concerning patient comprehension of the drug regimen were created among those patients who obtained AL at the health facility using questions regarding the duration of treatment (number of days), times per day the medicine was to be given, and dosage (number of tablets). The respondent’s answers were considered with respect to the correct dosage determined using patient age and weight information.

MIS indicators focus on treatment-seeking behaviour and outcomes among people with fever in the two weeks preceding the survey. This sample of recently febrile individuals included children under five years of age using reports from women age 15 to 49 about their own biological children as well as all household members from the household listing. While the 2012 MIS survey report focuses on fever treatment-seeking behaviour for children under five [21], this paper uses secondary data analysis to produce estimates for people of all ages. Indicators were calculated from the household listing for all household members, except in the case of children under five with information available from the mother’s report, which serves as the standard source of fever prevalence and treatment-seeking behaviour used by major household survey groups. Variables were created to indicate treatment seeking for recent fever at an 'appropriate provider’ – defined as any public or private health facility or a community health worker (CHW). Treatment-seeking and ACT treatment were calculated among all febrile individuals, however information on blood testing (recall of a finger or heel stick as a standard proxy measure for a malaria blood test) was only available for children under five recorded in the woman’s questionnaire.

Tabulations and cross tabulations were performed using Stata 12.1 (© StataCorp, College Station, TX, USA). Point estimates and 95% confidence intervals (CI) are reported. Data analyses took into account the survey design: MIS stratification (urban/rural) and clustering of households within standard enumeration areas, and HFS stratification (level of care) and clustering of patients within health facilities. All data were weighted to account for disproportionate allocation of the sample to different strata.

An overall measure of systems effectiveness was calculated across key indicators on the pathway from ACT efficacy to effectiveness as follows: systems effectiveness = [AL efficacy (documented 100% [26])] x [% people with current or recent fever who reportedly sought treatment from an appropriate provider (MIS)] x [% patients with current or recent febrile illness seeking care at an appropriate provider who received a malaria blood test (HFS)] x [% patients with a positive blood test who received AL (HFS)] x [% patients prescribed AL who demonstrated full comprehension of the treatment regimen (HFS, proxy for adherence)] [16].

## Results

### 2012 Malaria Indicator Survey (MIS)

**Figure 1:**
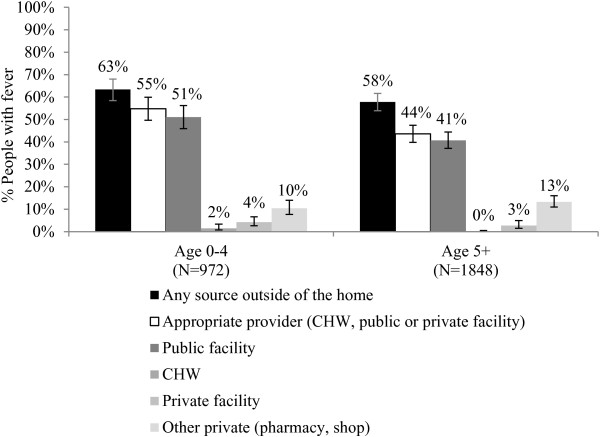
**Percentage of people with recent fever who reportedly sought treatment outside of the home, across age.** Source: MIS 2012 household roster and woman’s questionnaire.

The MIS captures self-reported fever management outcomes among people with fever in the two weeks prior to the household survey. More than half of children under five years of age (63%) and people age five years and above (58%) sought fever treatment outside of the home. Treatment from an appropriate provider (CHW, public health facility, or private health facility) was sought among more than half (55%) of children under five years and 44% of people age five years and above (Figure 1). Among people seeking treatment from an appropriate provider (N = 1,257), 71% reportedly visited a public health centre; 15% visited a public hospital; 4% visited a public health post; 1% visited a CHW; and 7% visited a private facility. Seeking fever treatment from other private outlets is reportedly uncommon in Zambia (10% of children under five and 13% of people age five and above) (Figure 1). Among people seeking treatment in other private outlets (N = 333), 71% reportedly visited a shop and 28% visited a pharmacy.

**Figure 2:**
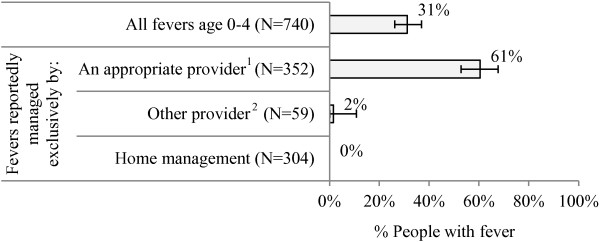
**Percentage of children under five with recent fever who reportedly received a blood test, across provider type.** Source: MIS 2012 woman’s questionnaire. 1 CHW, public or private facility. 2 Pharmacy, shop.

The MIS measured blood testing among children under five recorded in the woman’s questionnaire. Among children with recent fever, 31% reportedly received a blood test for malaria. Blood testing varied according to where children were taken for treatment. Among children managed exclusively by an appropriate provider, 61% received a blood test. Among children managed exclusively by a pharmacy or shop, only 2% reportedly had a blood test (Figure 2).

**Figure 3:**
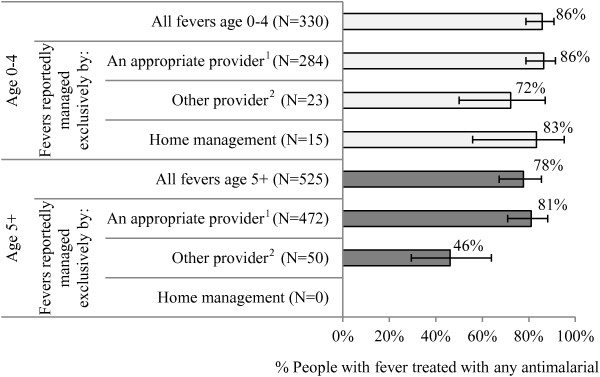
**Percentage of people with recent fever treated with an anti-malarial who received ACT treatment, across age and provider type.** Source: MIS 2012 household roster and woman’s questionnaire. 1 CHW, public or private facility. 2 Pharmacy, shop.

Figure 3 examines use of ACT relative to other anti-malarials reportedly used for recent fever. Among those reportedly treated with any anti-malarial, 86% of children under five and 78% of people age five and above received an ACT. ACT treatment was higher among people exclusively managed by an appropriate provider (86% of children under five, 81% of people age five and above) as compared with people managed by pharmacies or shops (72% of children under five, 46% of people age five and above) (Figure 3).

Malaria RDTs were administered and BS specimens for microcopy were collected from children under five who were recorded in the woman’s questionnaire. Among recently febrile children who did not seek treatment from an appropriate provider, current infection was identified in the 30% who were both BS and RDT positive. These results indicate that a substantial fraction of children with febrile illness who are not taken to an appropriate provider actually harbour malaria infection – an infection that may persist untreated, contributing to transmission, ongoing morbidity, and potentially severe illness and even death. About half of recently febrile children who did not seek treatment from an appropriate provider had no evidence of malaria infection (44% BS and RDT negative), and an additional 25% were BS negative and RDT positive – possibly indicating recent anti-malarial treatment with medications that were stored at home or acquired from another source (Table 1).

**Table 1:** Malaria infection among recently febrile children, across treatment-seeking behaviour and age

**Did not seek treatment from an appropriate provider**^**1**^						
	**All children**	**0-11 months**	**12-23 months**	**24-35 months**	**36-47 months**	**48-59 months**
N fever	443	62	95	85	113	88
% BS-, RDT-	44.1	70.3	44.3	53.4	32.2	32.2
	(36.1-52.5)	(57.8-80.3)	(31.2-58.2)	(38.5-67.8)	(22.1-44.2)	(20.3-46.9)
% BS+, RDT+	29.9	17.4	39.0	27.3	31.0	31.0
	(24.1-36.6)	(9.7-29.3)	(26.3-53.3)	(17.6-39.8)	(22.3-41.4)	(22.5-41.0)
% BS-, RDT+	24.8	9.4	16.8	19.3	34.7	35.7
	(19.4-31.0)	(3.4-23.4)	(11.0-24.8)	(11.5-30.5)	(25.8-44.7)	(23.7-49.8)
% BS+, RDT-	1.2	2.9	0.0	0.0	2.1	1.2
	(0.5-3.0)	(0.7-11.1)			(0.7-6.6)	(0.2-8.3)
**Sought treatment from an appropriate provider**^**1**^						
	**All children**	**0-11 months**	**12-23 months**	**24-35 months**	**36-47 months**	**48-59 months**
N fever	451	74	86	110	103	78
% BS-, RDT-	49.8	68.8	59.2	45.3	41.6	34.1
	(41.9-57.7)	(56.2-79.1)	(43.9-72.9)	(34.1-57.0)	(30.1-54.0)	(22.5-48.0)
% BS+, RDT+	16.2	6.1	13.1	21.9	16.4	23.4
	(12.6-20.7)	(2.9-12.3)	(7.7-21.4)	(14.4-32.0)	(10.7-24.4)	(14.3-35.9)
% BS-, RDT+	33.2	23.1	27.7	32.8	41.2	41.3
	(27.1-40.0)	(14.9-34.0)	(17.9-40.3)	(23.5-43.7)	(29.3-24.4)	(30.5-53.0)
% BS+, RDT-	0.7	2.1	0.0	0.0	0.8	1.2
	(0.3-2.0)	(0.5-8.1)			(0.1-5.8)	(0.2-8.0)

Source: MIS 2012 household roster and woman’s questionnaire.

1 CHW, public or private facility.

Among children who were taken to an appropriate provider for fever treatment, 16% remained BS and RDT positive at the time of the survey, and an additional 33% were BS negative and RDT positive (Table 1). The fraction of children who reportedly received care from an appropriate provider but nonetheless remained BS positive at the time of the survey suggests that these infections were not effectively managed.

Among children under five years who were BS and RDT positive, fewer than half (44%) reportedly experienced fever in the two weeks preceding the survey. Prevalence of recent fever among infected children declines with age from 61% to 37% between ages 12 to 23 months to age 48 to 59 months (Table 2).

**Table 2:** Recent fever among infected children, across age

	**N BS and RDT+**	**% Recent fever (95% CI)**
**All children**	**512**	**43.7 (36.8-50.9)**
Age		
0-11 months	46	39.0 (25.2-54.8)
12-23 months	85	61.2 (47.6-73.2)
24-35 months	122	43.4 (33.9-53.3)
36-47 months	124	42.4 (31.7-53.8)
48-59 months	135	36.5 (26.5-47.8)

Source: MIS 2012 household roster and woman’s questionnaire.

### 2011 Health Facility Survey (HFS)

**Figure 4:**
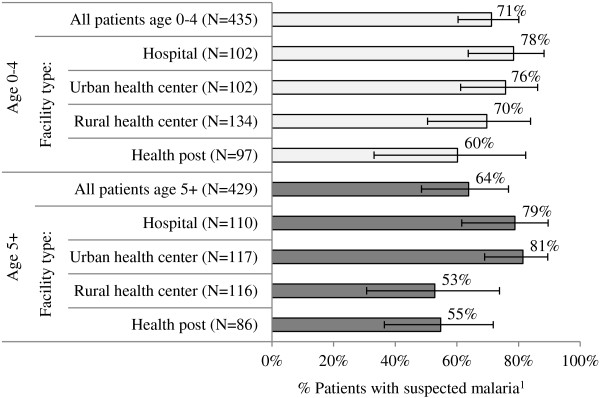
**Percentage of patients with suspected malaria**^**1 **^**who received a blood test for malaria, across level of care and patient age.** Source: HFS 2011. 1 Self-reported fever or a history of fever and/or body temperature greater than or equal to 37°C.

The HFS documents observed fever management outcomes among patients with suspected malaria (fever or history of fever) seeking treatment at a health facility. Among patients with suspected malaria exiting public health facilities in 2011, 71% of children under five years and 64% of people age five years and above received a blood test for malaria. Data trends show higher testing rates among patients managed in hospitals and urban health centres as compared with rural health centres and health posts (Figure 4).

**Figure 5:**
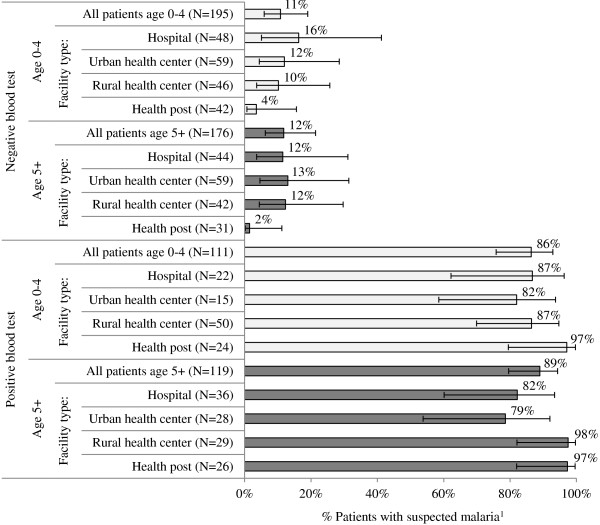
**Percentage of patients who were prescribed the first line ACT (AL) across blood test results, patient age, and level of care.** Source: HFS 2011. 1 Self-reported fever or a history of fever and/or body temperature greater than or equal to 37°C.

Among patients tested for malaria during the consultation, 230 (39%) tested positive and 378 (61%) tested negative. Among patients who tested positive during consultation, 86% of children under five and 89% of people age five and above were prescribed ACT. Low levels of ACT prescription were observed among patients that tested negative during consultation: 11% of children under five and 12% of people age five and above (Figure 5). Use of other anti-malarials (quinine, sulphadoxine-pyrimethamine (SP)) to treat suspected malaria cases was infrequently observed among all patients including those who were not tested during consultation (3%); patients with a negative blood test result (3%); and patients with a positive blood test result (3%).

**Figure 6:**
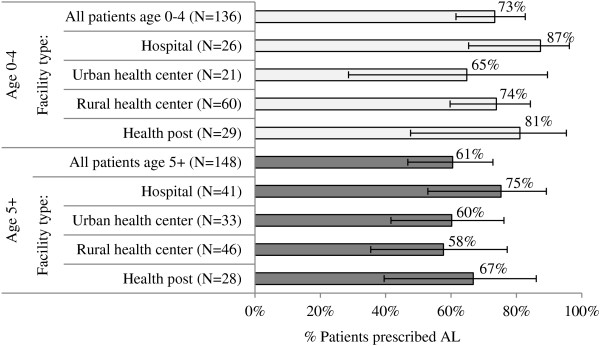
**Percentage of patients prescribed AL who demonstrated full comprehension of the drug regimen (duration, dosage, and number of times per day), across patient age and level of care.** Source: HFS 2011.

Patients exiting with a prescription for AL were asked to explain the drug regimen, including dosage (number of tablets based on age/weight), doses per day (two times), and number of days to give the drug (three days). Complete correct knowledge of the regimen was demonstrated by 73% of caregivers of children under five and 61% of caregivers/patients age five and above (Figure 6).

### Systems effectiveness for malaria case management

**Figure 7:**
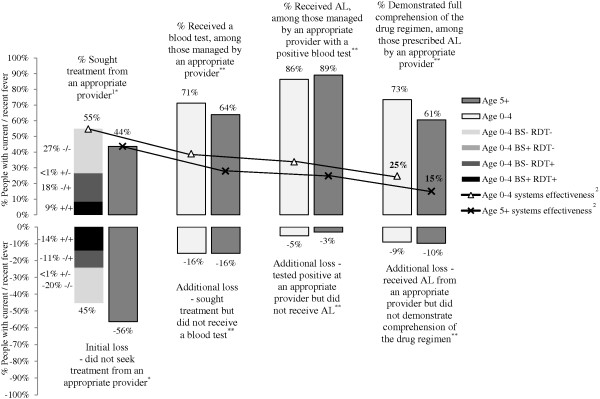
**Systems effectiveness for malaria case management in Zambia.** * Source: 2012 MIS, ** Source: 2011 HFS. 1 CHW, public or private facility. 2 Systems effectiveness = [ACT efficacy] x [sought fever treatment from an appropriate provider] x [malaria blood testing] x [AL prescription for positive cases] x [AL regimen comprehension]. The calculation for children under five is: [1.00] x [0.548] x [0.712] x [0.864] x [0.734] = 0.247, or 25%. The calculation for people age five and above is: [1.00] x [0.436] x [0.638] x [0.89] x [0.605] = 0.15, or 15%.

The overall systems effectiveness for malaria case management is 25% for children under five years and 15% for people age five years and above (Figure 7). These results are influenced most heavily by losses at the first step in the case management cascade – low rates of seeking treatment from an appropriate provider. More than half (56%) of people age five years and above and 45% of children under five years of age with suspected malaria (i.e. current or recent fever) are lost at this stage. Blood test results among children under five indicate that the loss is considerably smaller (14%) when considering only those children who had positive RDT and BS results (Figure 7).

## Discussion

Documenting malaria case management progress in the context of recent ACT and RDT scale-up has been a challenge given limitations of the most commonly available data sources - population-based household surveys (MIS, DHS) and HMIS data. The challenge to document progress in Zambia was addressed by applying a systems effectiveness approach using the available national household survey data (2012 MIS), and the recently available national health facility survey data (2011 HFS). Assembling results from these surveys, overall systems effectiveness was estimated at 25% for children under five, and 15% for people age five and above. In other words, malaria diagnosis and treatment services in Zambia are effective in managing 25% and 15% of suspected cases under age five and age five years and above, respectively. Tracking the steps from efficacy to systems effectiveness highlighted areas that require strengthening to improve case management coverage.

The first step in the cascade from ACT efficacy to systems effectiveness is seeking treatment from an appropriate provider. In this context, appropriate providers include public or private health facilities and CHWs. These sources are the focus of national policies, guidelines, and support for malaria case management. The definition of an appropriate provider should be modified according to the set of promoted providers in a given country. Failure to seek treatment from an appropriate provider represents the largest threat to systems effectiveness in Zambia; only about half of people with recent fever reportedly sought care from an appropriate provider. This gap suggests a need for more information on barriers and facilitating factors for fever treatment-seeking behaviour in this context. The MIS is well suited to estimating levels of and at least some of the factors determining treatment-seeking behaviour for recent fever. These include a measure of household socio-economic status, child characteristics including age, and mother’s education, malaria knowledge, and exposure to malaria communications, and household distance to a health facility or appropriate care provider. This information can be used to improve intervention targeting.

To address gaps in treatment-seeking behaviour, MIS and other population-based data on treatment-seeking behaviour should be reviewed in the context of national programme information on service availability. For example, partners in Zambia have focused resources on improving access to malaria case management services through extension of diagnosis and treatment to the community level. CHWs are recruited, trained and supervised to provide malaria-specific or integrated packages of community-based case management [27]. Results from the 2012 MIS suggest that, at national level, fever cases are not typically managed by these community agents (only 2% of children under five with recent fever). However, given that community case management programme coverage is subnational, additional analyses should evaluate CHW service utilization explicitly in areas with current programme coverage. These analyses would provide information on the extent to which the strategy facilitates appropriate treatment-seeking behaviour. Assembling existing data on service availability and utilization can facilitate targeted investment in scale-up to improve national coverage. Where existing data cannot adequately explain underutilization of accessible care options, additional information about treatment-seeking behaviour may be needed. Qualitative research, often entailing use of rapid ethnographic methods, is commonly employed to understand the complexities and variations in illness identification and treatment-seeking behaviour. Recent reviews suggest that such studies could be strengthened through application of theory and longer-term engagement through methods such as extended case studies and ethnography [28-30].

The second systems effectiveness step is blood testing for suspected malaria. Frequency of blood testing as measured in the Zambia HFS was high; 71% of patients under age five and 64% of patients age five years and above received a blood test. The MIS also provides a rough approximation of blood testing in this context and suggests that 61% of children under five taken to an appropriate provider were tested for malaria. Findings from a recent Zambia study to validate standard MIS questions also suggest that the MIS may underestimate blood testing [9]. While both surveys show impressive improvement in malaria testing coverage in Zambia [31,32], gaps persist. Health facility assessments provide an opportunity for measuring a number of factors that may influence blood-testing practices; these include facility factors (e g, availability of RDTs, microscopy, national guidelines, and job aids, level of care, staffing), provider factors (e g, training, supervision, experience, satisfaction, client case load), and patient factors (e g, age, other symptoms).

The third essential step for systems effectiveness is ACT treatment for people with confirmed malaria infection. Zambia HFS results indicate tremendous progress in this area - most people who test positive for malaria are prescribed ACT (86% for children under five and 89% for age five and above). The HFS is unique in its ability to determine rates of ACT prescription based on test results. Additionally, HFS results in Zambia indicate that in most cases, ACT is dispensed rationally based on test results; ACT was prescribed to just one in ten patients who tested negative for malaria. Tracking rational use can help explain reductions in systems effectiveness at this stage where over prescription may contribute not only to inefficiencies, but to stock-outs that ultimately prevent ACT prescription for positive cases. ACT use based on test results is not well captured in household surveys. The MIS can include questions about malaria blood test results, however patients seeking care may not be adequately and accurately informed about test results and/or may not accurately recall the result [9]. Routine collection of facility data through the HMIS could capture information on testing and treatment. However, in the Zambian context, the HMIS collects aggregate reports on numbers of patients tested and treatments dispensed, rather than individual patient data on testing, diagnosis, and treatment. Efforts to strengthen the reporting and linkages of data on stock management and disease reporting could facilitate additional triangulation of information for review on a routine basis.

With the wide recognition that the previous global indicator based on presumptive anti-malarial treatment of fever is no longer informative, a new indicator proposed by the Roll Back Malaria Monitoring and Evaluation Reference Group is “the proportion of people receiving an ACT, among people with fever in the last two weeks who received any anti-malarial drugs [7]”. Applying this indicator in Zambia, most anti-malarial treatments reportedly received are ACT (86% for children under five and 78% for age five and above), particularly where treatment is acquired from an appropriate provider. In other contexts, where the anti-malarial market is diverse both in terms of market share and in types of anti-malarials in circulation, this MIS indicator could provide information on anti-malarial prescription among various provider types to supplement a more targeted facility survey focused on tracking behaviour among certain providers. Nonetheless, the MIS ACT indicator denominator is only among those individuals who received any anti-malarial and does not permit evaluation of those not receiving an anti-malarial for whatever reason. The HFS can track appropriate and rational ACT use, and can capture and explore facility, provider, and patient factors associated with prescription behaviour to inform intervention.

The final step in the systems effectiveness framework is patient adherence to the drug regimen. Data on drug adherence are often imperfect; the behaviour is difficult to measure, self-report measures may be unreliable [33], and a 2005 review noted that there is insufficient information on adherence to anti-malarials [34]. In the absence of a direct measure of adherence to AL in Zambia, a proxy measure was used - comprehension of the drug regimen at exit from a health facility. While the relationship between comprehension and adherence may not be exact, a recent study of AL adherence in Kenya identified correct knowledge of the drug regimen as the strongest predictor of adherence [35]. To fully estimate effective case management intervention coverage, and to monitor behaviours that can contribute to anti-malarial drug resistance, additional study designs and instruments are needed.

The systems effectiveness approach to assemble household and facility data appears to provide the most complete picture of malaria case management to date. However, one of the limitations in linking these data sources through this approach is in treating self-reported fever as a proxy and denominator for suspected malaria in the overall calculation of systems effectiveness. The approach assumes that febrile individuals with malaria infection are no more or less likely to seek treatment than febrile individuals who do not harbour malaria infection. Following this logic, all fevers must be managed by an appropriate provider in order to reach all malaria cases. In reality, the malaria-attributable fraction of febrile cases varies across seasons and contexts, and declines with scale-up of control interventions [36]. In treating all reported fevers the same, the approach underestimates the overall systems effectiveness for managing true symptomatic parasitaemic cases. Indication of this under-estimation is apparent in examining blood test results for children with fever who were not taken to an appropriate provider; nearly half were both BS and RDT negative. It is also important to note that this framework is limited in its application to management of *symptomatic* malaria infections. Focusing on symptomatic cases to reduce severe disease and death is appropriate in the context of malaria control in high transmission settings. However, asymptomatic (afebrile) infections contribute to ongoing transmission as well as disease burden [37,38]. Analysis of MIS blood test results among children under five indicates that fewer than half (44%) of children that were both BS and RDT positive reportedly experienced recent fever. Clearing malaria infections – both symptomatic and asymptomatic – will require different intervention strategies and different metrics to document progress [39-41].

This study used standard household and facility survey instruments and methods, and data were collected among a nationally representative sample. Despite these strengths, each data source has limitations. First, while each study provided reasonably precise estimates for key indicators, comparisons across age groups and levels of care resulted in loss of precision (i e, wide confidence intervals) due to smaller sample sizes among these subgroups. Sample size calculations for facility surveys are challenging given a number of factors that influence the ultimate numbers of study participants among each subgroup of interest, including cases of suspected malaria, confirmed cases, and treated cases. Measurement bias is another limitation in both studies. Household surveys estimate aspects of coverage using respondent reports, which are subject to recall bias. This bias is minimized in the MIS and other standard surveys by administering questions regarding fever that occurred recently - in the two weeks prior to the survey. Initial studies aimed at validating respondent recall and response suggest need for further research [9]. Consultation observation and patient exit interviews were used in the HFS. These methods are subject to the Hawthorn effect – a bias that may arise due to study participant awareness of being under observation; health workers may perform better than usual given presence of study teams [42,43]. This study facilitated triangulation of HFS results with MIS findings to some extent (e g, regarding blood testing for suspected malaria), thereby strengthening conclusions. Neither data source provided direct measure of adherence to AL – a major limitation to completing the systems effectiveness framework. Further research is needed on this behaviour in Zambia. Data to estimate systems effectiveness were collected through two discrete surveys, one in 2011 (HFS) and one in 2012 (MIS). Temporal differences are important to consider when assembling data from different surveys because in many contexts, case management interventions and coverage are rapidly evolving. While data from Zambia used in this analysis were not collected simultaneously, the systems they were evaluating were relatively stable over the time period encompassing both surveys, facilitating meaningful combination of survey results. While the systems effectiveness framework highlighted the extent to which gaps exist in the pathway from efficacy to effectiveness, there remains a need to delve deeper into each step to inform interventions. Specific strategies for improving treatment-seeking behaviour, as well as blood testing, rational AL use, and patient adherence, can be developed based on further studies of factors associated with these outcomes.

Applications of the systems effectiveness framework to benchmark progress in other country contexts may require additional data beyond what was applied in this study. Investments to improve malaria case management in Zambia have focused primarily on public health facilities and CHWs, augmented by a relatively small contingent of private health facilities. Among this set of appropriate providers, 2012 MIS data indicate that health facilities were commonly accessed while CHWs were an infrequent source of care for fever management at national level. Thus fever management components of the systems effectiveness framework were completed using information from health facilities. In comparison, health systems for case management in other malaria endemic countries are more diverse; appropriate providers who are authorized, trained, and promoted to manage malaria may include private sector providers including pharmacies, drug shops, and mobile vendors [44,45]. Although the health system for malaria case management is unique in each country, benchmarking progress using a systems effectiveness framework seems applicable across contexts. Where the system is comprised of multiple actors operating in community, public, and private spheres, assembling a picture of systems effectiveness will require information first on the extent to which each component of the system is accessed for care; and second, information on case management (blood testing and drug prescription) within each of the various system components. Ultimately, the utility of the systems effectiveness framework lies in the simplicity of assembling and interpreting data from multiple sources. This utility should be tested in the context of diverse health systems for malaria case management.

## Conclusion

Health facility and population-based household survey data (HFS and MIS) applied to the systems effectiveness framework provides a cogent and feasible approach to documenting malaria case management coverage. In Zambia, these results indicate that the largest threat to systems effectiveness is appropriate treatment-seeking behaviour – which can encompass both access to appropriate case management service providers as well as service utilization. Although room for improvement remains, study results demonstrate high facility coverage of blood testing for patients with symptoms of malaria and high rates of rational ACT use. Further analysis of the MIS and HFS can identify specific factors associated with treatment-seeking behaviour, blood testing, and rational AL use and can inform interventions to improve malaria diagnosis and treatment. Until now, tracking progress in malaria case management coverage has relied heavily on population-based surveys. The way forward likely entails household surveys to track trends in treatment-seeking behaviour, and facility/provider data (survey, routine, or supervisory data) to track appropriate management of suspected malaria cases. Finally, as programmes embark on the road to malaria elimination, blood-testing components of household surveys can document the extent to which infections are symptomatic (febrile) and recent fever is an indicator of infection. This information can shape appropriate interventions to target both symptomatic and asymptomatic infections.

## Competing interests

The authors declare that they have no competing interest.

## Authors’ contributions

JMM and MN designed and implemented the 2011 HFS. JMM led the fieldwork and data analysis for the 2012 MIS. MH led the laboratory work for the 2012 MIS. ML and RWS conceived the approach to assembling the data. ML performed the data analysis and wrote the first draft of the manuscript. ML, JMM, MN, BH, MH, MK, DHH, and RWS contributed to writing of the manuscript. All authors read and approved the final version of the manuscript.
